# Higher Fibrinogen Level is Independently Linked with the Presence and Severity of New-Onset Coronary Atherosclerosis among Han Chinese Population

**DOI:** 10.1371/journal.pone.0113460

**Published:** 2014-11-26

**Authors:** Yan Zhang, Cheng-Gang Zhu, Yuan-Lin Guo, Rui-Xia Xu, Sha Li, Qian Dong, Jian-Jun Li

**Affiliations:** Division of Dyslipidemia, State Key Laboratory of Cardiovascular Disease, FuWai Hospital, National Center for Cardiovascular Diseases, Chinese Academy of Medical Sciences, Peking Union Medical College, BeiLiShi Road 167, Beijing, 100037, China; University Heart Center Freiburg, Germany

## Abstract

**Background:**

Fibrinogen is a coagulation/inflammatory biomarker strongly associated with atherogenesis. However, no data is currently available regarding the association of fibrinogen level with the presence and severity of new-onset coronary atherosclerosis assessed by Gensini score (GS), particularly in Han Chinese with a large sample size.

**Methods and Results:**

We studied 2288 consecutive, new-onset subjects undergoing coronary angiography with angina-like chest pain. Clinical and laboratory data were collected. Coronary stenotic lesions were considered to be the incidence of coronary atherosclerosis. The severity of coronary stenosis was determined by the GS system. Data indicated that patients with high GS had significantly elevated fibrinogen level (p<0.001). The prevalence and severity of coronary atherosclerosis were dramatically increased according to fibrinogen tertiles. Spearman correlation analysis revealed a positive association between fibrinogen level and GS (r = 0.138, p<0.001). Multivariate logistic regression analysis demonstrated that plasma fibrinogen level was independently associated with high GS (OR = 1.275, 95% CI 1.082–1.502, p = 0.004) after adjusting for potential confounders. Moreover, fibrinogen level was also independently related to the presence of coronary atherosclerosis (fibrinogen tertile 2: OR = 1.192, 95% CI 0.889–1.598, p = 0.241; tertile 3: OR = 2.003, 95% CI 1.383–2.903, p <0.001) and high GS (fibrinogen tertile 2: OR = 1.079, 95% CI 0.833–1.397, p = 0.565; tertile 3: OR = 1.524, 95% CI 1.155–2.011, p = 0.003) in a dose-dependent manner. Receiver-operating characteristic curve analysis showed that the best fibrinogen cut-off value for predicting the severity of coronary stenosis was 3.21 g/L.

**Conclusions:**

Higher fibrinogen level is independently linked with the presence and severity of new-onset coronary atherosclerosis in Han Chinese population.

## Introduction

Coronary atherosclerosis is the major cause of associated morbidity and mortality in the world. The critical coronary artery stenosis is a strong predictor for cardiovascular adverse events [1]. Current perspectives regard thrombotic process and low grade chronic inflammation as critical contributors for plaque instability and stenosis progression [2],[3]. Identifying valuable and noninvasive markers is important for clinical decision-making in the treatment and prevention of coronary atherosclerosis.

Fibrinogen is by far the most abundant coagulation factor in the blood. In addition to its essential properties as a cofactor of platelet aggregation and as part of the final common pathway of the coagulation cascade, fibrinogen is also a well-known acute phase protein [4]. During the last decades, several studies focused on fibrinogen and its relation to high risk of atherosclerotic diseases, most of which demonstrated that fibrinogen played a pivotal role in the initial phase and progressive stages of atherosclerosis [5],[6]. Multiple epidemiologic along with case-control studies revealed an association between the increased fibrinogen level and the early signs of atherosclerosis in asymptomatic individuals [7],[8]. The increased fibrinogen level has also been identified as an important risk factor for the future cardiovascular events in apparently healthy individuals [9],[10] and in patients with established coronary artery disease (CAD) [11]–[13]. Therefore, fibrinogen is proposed as a potential predictor for the risks of cardiovascular diseases.

Although much positive evidence has been identified, more recent studies showed that fibrinogen could not provide additional information to that provided by traditional cardiovascular risk factors in predicting cardiovascular events [14]. Additionally, according to fibrinogen genetic studies, polymorphisms which related to fibrinogen level were not associated with an increased cardiovascular risk [15]. Therefore, the clinical significance of fibrinogen in the risk stratification for cardiovascular disease is still controversial. Besides, previous reports investigated the relation between fibrinogen and the severity of coronary atherosclerosis in patients with chronic CAD or acute coronary syndrome. Data enrolled patients with new-onset coronary atherosclerosis quantitatively by coronary angiography and Gensini score (GS) system were obscure. Moreover, large-scale studies were currently unavailable regarding subjects representative of Han Chinese population.

Therefore, the aim of the current study was to investigate the association of plasma fibrinogen level with the presence and severity of new-onset coronary atherosclerosis assessed by GS in a large cohort of Han Chinese population.

## Materials and Methods

### Study design and population

The study protocol complied with the Declaration of Helsinki, and was approved by the hospital ethics review board (FuWai Hospital & National Center for Cardiovascular Diseases, Beijing, China). Informed written consent was obtained from all patients included in this study.

This was a hospital-based prospective and observational study. From April 2011 to March 2014, we consecutively enrolled 2288 new-onset subjects referred to elective coronary angiography with angina-like chest pain in our institution. Complete medical history was taken from all subjects. Patients with new-onset coronary atherosclerosis were diagnosed by physician for the first time based on coronary angiography or typical chest pain without prior history of coronary atherosclerosis. Coronary stenosis was assessed by at least two independent senior interventional cardiologists [16].

Exclusion criteria were patients with no fibrinogen measurements available, emergency admission, the existence of any life-threatening arrhythmias, cardiac valve disease, previous history of coronary atherosclerosis, heart failure (The left ventricular ejection fraction (LVEF) <45%), infectious or systematic inflammatory disease, significant hematologic disorders, thyroid dysfunction, severe liver and/or renal insufficiency, and malignant tumors.

### Definition of traditional cardiovascular risk factors

Main cardiovascular risk factors were defined using standard criteria. Hypertension was defined as repeated systolic and/or diastolic blood pressure ≥140 and/or ≥90 mmHg on two different occasions or if patients were currently taking anti-hypertensive drugs. Diabetes mellitus was diagnosed as fasting serum glucose levels ≥7.0 mmol/L in multiple determinations or was receiving hypoglycemic treatments (dietary, oral anti-diabetic agents, or insulin). Dyslipidemia was defined by medical history or the use of lipid-lowering medications in order to reduce lipids or fasting total cholesterol (TC) ≥5.18 mmol/L or triglyceride (TG) ≥1.70 mmol/L. Smoking status was ascertained by the medical history.

### Biochemical analyses

Fasting blood samples were obtained at baseline from each patient. The plasma fibrinogen level was quantitatively measured by the method of Clauss [17] and a Stago auto analyzer with STA Fibrinogen kit (Diagnostic Stago, Taverny, France). The concentration of high sensitivity C-reactive protein (hs-CRP) was determined using immunoturbidimetry (Beckmann Assay 360, Bera, Calif., USA). White blood cell count (WBCC) was determined by an automated blood cell counter (Beckman Coulter Ireland Inc Mervue, Galway, Ireland). The concentrations of Lipid profiles were determined by automatic biochemistry analyzer (Hitachi 7150, Tokyo, Japan). In detail, the low-density lipoprotein-cholesterol (LDL-C) concentration was analyzed by selective solubilization method (Low density lipid cholesterol test kit, Kyowa Medex, Tokyo). High-density lipoprotein cholesterol (HDL-C) concentration was determined by a homogeneous method (Determiner L HDL, Kyowa Medex, Tokyo). TC, TG, apolipoprotein A1 (apoA1), apolipoprotein B (apoB), and lipoprotein (a) [Lp(a)] were measured with commercial kits.

### Severity of coronary atherosclerosis

The severity of coronary atherosclerosis was quantified by GS system [18],[19]. The GS was computed by assigning a severity score to each coronary stenosis according to the degree of luminal narrowing and the importance of location, which was defined as 1 point for stenosis of 1–25%, 2 points for 26–50%, 4 points for 51–75%, 8 points for 76–90%, 16 points for 91–99% and 32 points for total occlusion. The score was then multiplied by a factor that represented the importance of the lesion's position. That was 5 for the left main coronary artery, 2.5 for the proximal left anterior descending or proximal left circumflex artery, 1.5 for the mid-region, 1 for the distal left anterior descending or mid-distal region of the left circumflex artery, and 0.5 for small vascular branches. In patients who have undergone revascularization, the angiographic severity was measured before the procedures.

### Statistical analysis

Quantitative variables were expressed as mean ± standard deviation (SD) or median with interquartile range (IQR), and were analyzed by Student's t tests, one way ANOVA, Mann-Whiteney U tests or Kruskal-Wallis tests as appropriate. Qualitative variables were expressed as numbers and percentages, and were assessed by chi-squared tests. The correlation between plasma fibrinogen level and GS was examined by Spearman correlation analysis. Univariate and multivariate logistic regression analyses were performed to determine the association of fibrinogen and other risk factors with the presence and severity of coronary atherosclerosis. Receiver operating characteristics (ROC) curves were constructed to document the predictive value of fibrinogen and other markers for high GS. A p value of less than 0.05 was considered statistically significant. Statistical studies were carried out with the SPSS program (version 19.0, SPSS, Chicago, Illinois, USA).

## Results

### Baseline clinical characteristics

**Figure 1:**
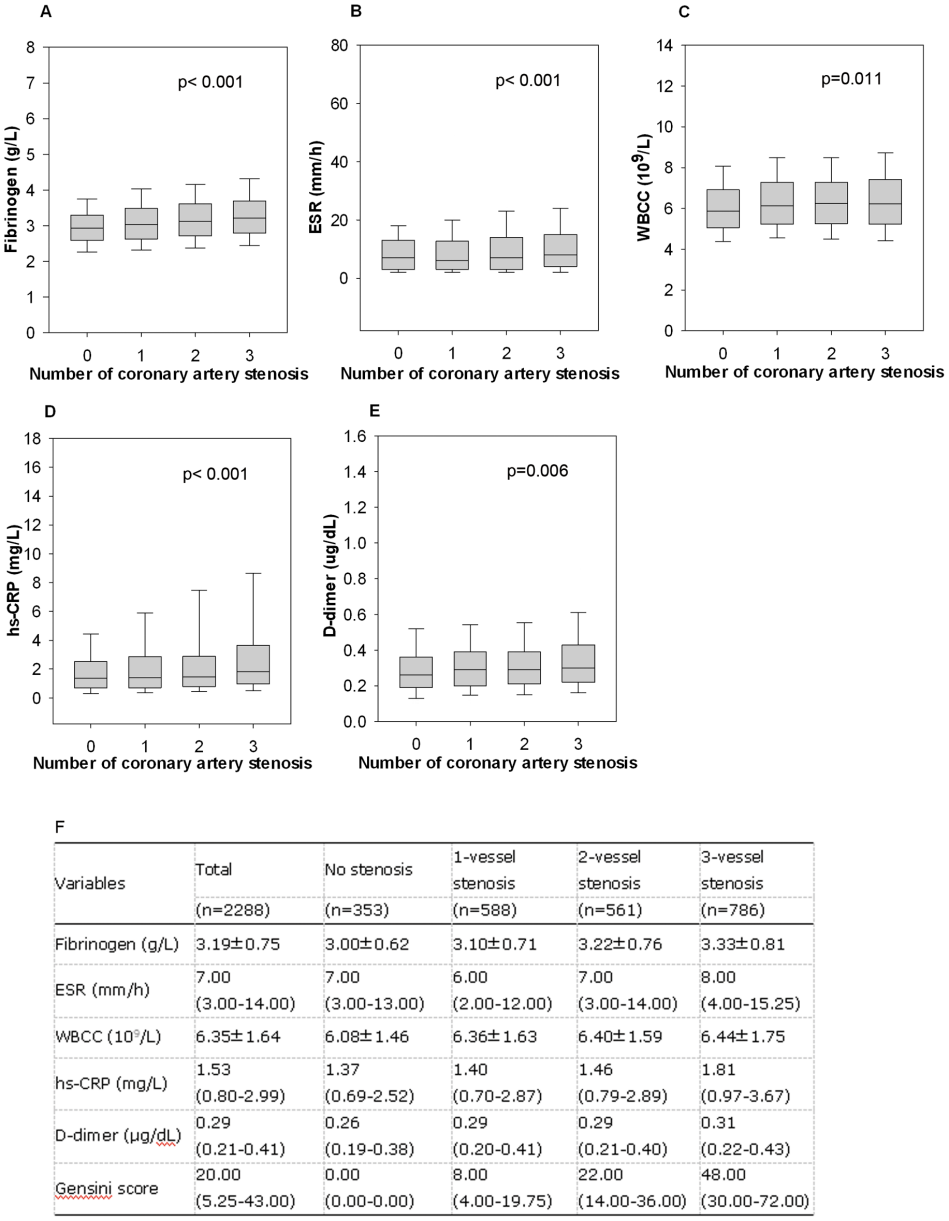
The levels of fibrinogen and other non-specific inflammatory markers according to the numbers of stenotic vessels (A: Fibrinogen; B: ESR; C: WBCC; D: hs-CRP; E: D-Dimer; F: Table shows data from figure above, presented as mean± SD or median (25th-75th percentile)). ESR = Erythrocyte sedimentation rate; WBCC = White blood cell count; hs-CRP = High sensitivity C-reactive protein.

The study population consisted of 2288 eligible subjects referred to coronary angiography with an average age of 57.8±9.9, and 62.4% of this study population was male. The majority of these subjects (n = 1935, 84.57%) had demonstrable coronary stenosis, whereas the remaining 353 (15.43%) subjects had no evidence of stenosis. Among patients with coronary stenosis, GS ranged from 1 to 200 (median 25). The baseline demographic, clinical characteristics and laboratory data of the enrolled subjects according to coronary stenosis and GS tertiles (no stenosis (n = 353); low GS (<16, n = 622); intermediate GS (16–39, n = 663); high GS (>39, n = 650) were summarized in Table 1. In brief, patients with high GS had higher percentage of smoking, hypertension, dyslipidemia and DM, higher level of TC, LDL-C, Lp(a), apoB, fasting blood glucose, hemoglobin A1C (HbA1C), and lower levels of HDL-C, apoA1 and LVEF. Meanwhile, the easily available inflammatory biomarkers such as fibrinogen, hs-CRP, WBCC, and erythrocyte sedimentation rate (ESR) levels were all markedly elevated according to GS tertiles (Table 1) as well as numbers of stenotic vessels (Figure 1).

**Table 1:** Comparison of baseline characteristics according to the presence and severity of coronary atherosclerosis.

			Gensini scores		
Variables	No stenosis	Coronary stenosis	Low	Intermediate	High
	(n = 353)	(n = 1935)	(<16; n = 622)	(16∼39; n = 663)	(>39; n = 650)
**Risk factors**					
Age (years)	54.84±9.41	58.33±9.89^b^	57.73±9.93	58.13±9.65	59.01±10.01^c^
Male, n (%)	186 (52.69)	1383 (71.47)^b^	406 (65.27)	475 (71.64)	502 (77.23)^d^
BMI (kg/m^2^)	25.78±3.66	25.62±3.17	25.37±3.04	25.71±3.34	25.78±3.11^c^
Current smoking, n (%)	132 (37.39)	1024 (52.92)^b^	309 (49.68)	367 (55.35)	348 (53.54)
Hypertension, n (%)	200 (56.66)	1221 (63.10)^a^	383 (61.58)	404 (60.94)	434 (66.77)
Dyslipidemia, n (%)	236 (66.86)	1460 (75.45)^b^	481 (77.33)	492 (74.21)	487 (74.92)
Diabetes mellitus, n (%)	44 (12.46)	528 (27.29)^b^	115 (18.49)	193 (29.11)	220 (33.85)^d^
Family history of CAD, n (%)	48 (13.60)	324 (16.74)	87 (4.9)	123 (18.92)	114 (17.54)
**Lipid profiles**					
TG (mmol/L)	1.49 (1.06–2.09)	1.54 (1.15–2.17)	1.50 (1.09–2.13)	1.55 (1.15–2.14)	1.58 (1.17–2.26)
TC (mmol/L)	4.41±0.94	4.26±1.15^b^	4.18±1.02	4.23±1.22	4.35±1.19^d^
LDL-C (mmol/L)	2.61±0.79	2.53±0.91	2.47±0.86	2.48±0.87	2.65±0.99^d^
HDL-C (mmol/L)	1.17±0.32	1.07±0.28^b^	1.10±0.28	1.07±0.29	1.05±0.26^d^
LP(a) (mg/L)	135.93 (58.64–304.80)	164.77 (68.76–377.44)^b^	158.59 (66.58–362.92)	154.87 (65.99–354.68)	183.41 (75.38–423.98)^c^
FFA (mmol/L)	0.41±0.19	0.41±0.19	0.39±0.18	0.42±0.18	0.42±0.20^c^
ApoA1 (g/L)	1.49±0.29	1.42±0.26^b^	1.44±0.26	1.41±0.26	1.39±0.25^d^
ApoB (g/L)	0.98±0.28	0.96±0.30	0.93±0.28	0.94±0.29	1.02±0.33^d^
**Clinical and laboratory test**					
LVEF (%)	65.62±5.11	64.55±6.01^b^	65.55±5.18	64.56±6.05	63.57±6.78^d^
HbA1c (%)	5.93±0.62	6.42±1.14^b^	6.21±0.97	6.45±1.19	6.60±1.21^d^
Glucose (mmol/L)	5.16±1.18	5.68±1.72^b^	5.52±1.62	5.69±1.80	5.82±1.72^d^
Platelet count (10^9^/L)	206.89±48.70	203.12±54.44	201.49±51.87	241.6±55.38	203.61±55.90
hs-CRP (mg/L)	1.37 (0.69–2.52)	1.59 (0.83–3.14)^b^	1.48 (0.75–2.88)	1.53 (0.78–3.26)	1.77 (0.95–3.33)^d^
WBCC (10^9^/L)	6.08±1.46	6.40±1.67^b^	6.25±1.60	6.42±1.71	6.53±1.68^d^
ESR (mm/h)	7 (3–13)	7.00 (3–14)^a^	7 (3–12)	7 (3–15)	8 (4–15)^d^
D-dimer (µg/dL)	0.26 (0.19–0.38)	0.29 (0.21–0.41)^b^	0.29 (0.21–0.41)	0.29 (0.21–0.40)	0.30 (0.21–0.43)
Fibrinogen (g/L)	3.00±0.62	3.23±0.77^b^	3.10±0.71	3.24±0.77	3.33±0.81^d^
**Prior treatment**					
Aspirin, n (%)	254 (71.96)	1741 (89.97)^b^	541 (87.03)	605 (91.23)	595 (91.53)
Beta-blocker, n (%)	139 (39.42)	940 (48.58)	263 (42.24)	368 (55.48)	309 (47.54)
ACEI/ARB, n (%)	89 (25.24)	431 (22.27)	136 (21.93)	158 (23.84)	137 (21.01)
Statin, n (%)	145 (41.06)	1195 (61.76)^b^	356 (57.28)	439 (66.25)	400 (61.61)

Data are expressed as n (%), mean± SD or median (25th–75th percentile). BMI =  Body mass index; CAD =  Coronary artery disease; TG =  Triglyceride; TC =  Total cholesterol; LDL-C =  Low-density lipoprotein-cholesterol; HDL-C =  High-density lipoprotein-cholesterol; LP(a) =  Lipoprotein(a); FFA =  Free fatty acid; ApoA1 =  Apolipoprotein A1; ApoB =  Apolipoprotein B; LVEF  =  Left ventricular ejection fraction; HbA1c =  Glycosylated hemoglobinA1C; hs-CRP =  High sensitivity C-reactive protein; WBCC =  White blood cell count; ESR =  Erythrocyte sedimentation rate; ACEI =  Angiotensin converting enzyme inhibitors; ARB =  Angiotensin receptor blocker.

a p<0.05 for coronary stenosis vs. no stenosis.

b p<0.01 for coronary stenosis vs. no stenosis.

c p<0.05 for high GS vs. low GS.

d p<0.01 for high GS vs. low GS.

### Association of fibrinogen with the presence and severity of coronary atherosclerosis

To explore the association of fibrinogen level with the presence and severity of coronary atherosclerosis, we classified the subjects into three groups according to fibrinogen tertiles. As shown in table 2, the prevalence of coronary atherosclerosis was significantly increased from 80.50% in tertile 1 to 90.34% in tertile 3 group (p<0.001). Moreover, the severity of coronary atherosclerosis assessed by the extent of stenosis, numbers of stenotic vessels or GS was significantly increased according to the tertiles of plasma fibrinogen level (all p<0.01).

**Table 2:** The presence and severity of coronary atherosclerosis according to fibrinogen tertiles.

	Total subjects	Fibrinogen tertiles (g/L)			p-value
	n = 2288	Tertile 1 (<2.83) n = 759	Tertile 2 (2.83–3.38) n = 756	Tertile 3 (>3.38) n = 766	
Age (years)	57.8±9.9	56.1±9.7	57.5±9.8	59.7±9.9	**<0.001**
Gender					**<0.001**
Female, n (%)	718 (31.38)	183 (24.11)	244 (32.28)	284 (37.08)	
Male, n (%)	1570 (68.62)	576 (75.89)	512 (67.72)	482 (62.92)	
BMI (kg/m^2^)	25.64±3.25	25.46±3.27	25.63±3.19	25.84±3.28	0.072
Coronary atherosclerosis, n (%)	1935 (84.57)	611 (80.50)	632 (83.60)	692 (90.34)	**<0.001**
Stenosis <50%, n (%)	178 (7.78)	69 (9.09)	56 (7.41)	53 (6.92)	0.089
Stenosis 50–75%, n (%)	277 (12.11)	93 (12.25)	106 (14.02)	78 (10.18)	**0.002**
Stenosis ≥75%, n (%)	1480 (64.68)	449 (59.16)	470 (62.17)	561 (73.24)	**0.002**
Number of stenotic vessels	2.10±0.84	1.99±0.84	2.10±0.84	2.22±0.82	**<0.001**
Gensini scores	25 (12–48)	22 (10–42)	24 (12–45)	28 (14–56)	**<0.001**

Data are expressed as n (%), mean± SD or median (25th–75th percentile). Bold values indicate statistical significance. BMI =  Body mass index.

**Figure 2:**
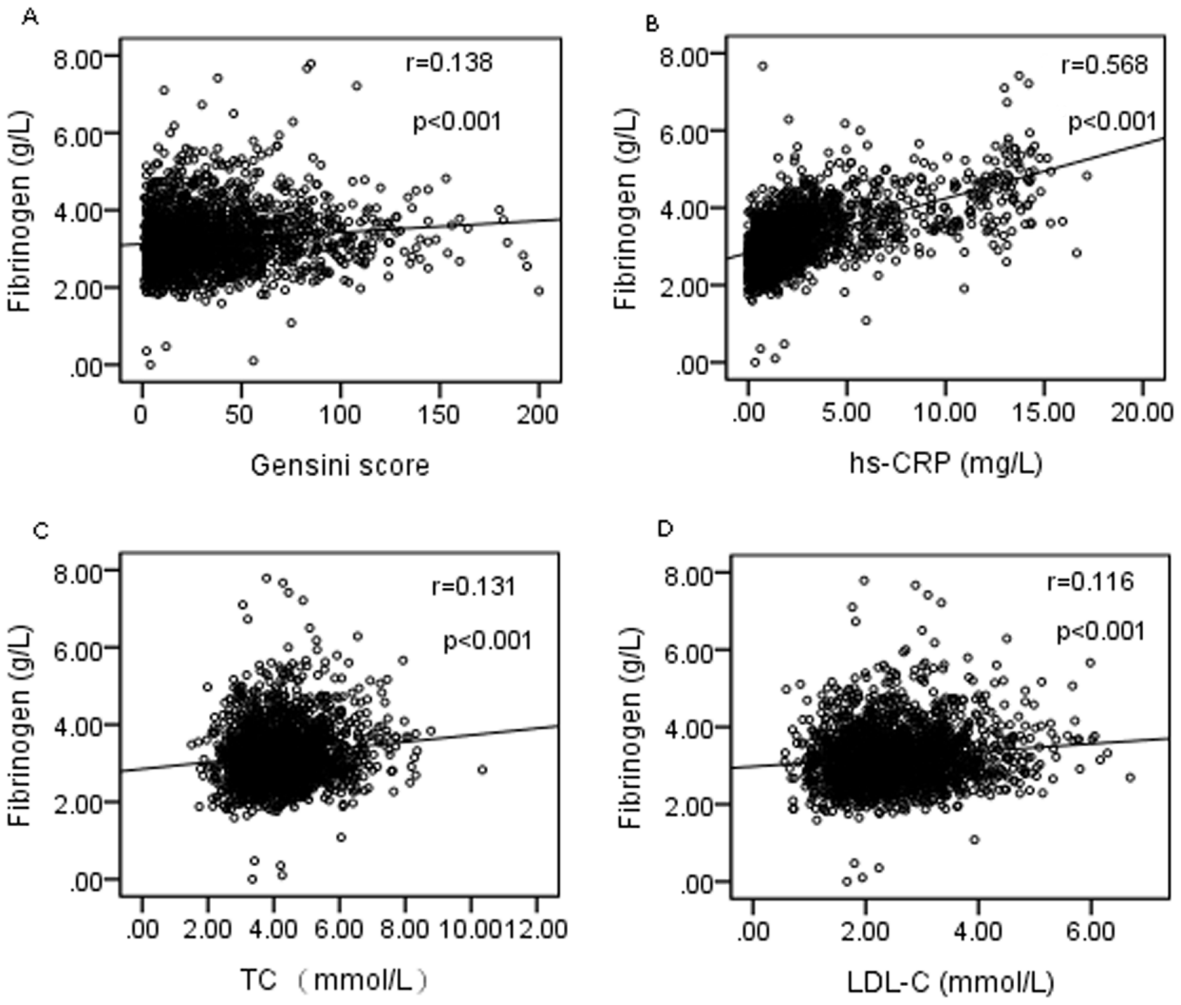
The correlations of fibrinogen level with Gensini score, hs-CRP, TC and LDL-C concentrations in patients with coronary atherosclerosis (A: Gensini score; B: hs-CRP; C: TC; D: LDL-C). hs-CRP =  High sensitivity C-reactive protein; TC =  Total cholesterol; LDL-C =  Low-density lipoprotein -cholesterol.

To further explore the relationship of plasma fibrinogen level with GS, Spearman correlation analysis was performed in the current study. As shown in Figure 2A, there existed a definitely positive correlation between plasma fibrinogen level and GS in patients with coronary atherosclerosis (r = 0.138, p<0.001). We additionally evaluated the relation of fibrinogen to other risk factors using the Spearman or Pearson correlation analysis as appropriate. The data revealed that plasma fibrinogen level was positively correlated with hs-CRP, TC or LDL-C concentrations (r = 0.568, p<0.001; r = 0.131, p<0.001, and r = 0.116, p<0.001, respectively; Figure 2B–D).

### Independence of fibrinogen level in predicting high GS

As indicated in Table 3, univariate and multivariate logistic regression analyses were performed to evaluate the value of plasma fibrinogen level in predicting high GS. After adjusting for gender, age, body mass index, current smoking, hypertension, family history of CAD, fasting blood glucose, TG, LDL-C, HDL-C and hs-CRP, plasma fibrinogen level was proved to be an independent predictor of high GS (OR = 1.275, 95% CI 1.082–1.502, p = 0.004). Additionally, multivariate logistic regression models also confirmed the independence of classic and new-emerging risk factors (ESR OR = 1.023, 95% CI 1.012–1.035, p<0.001; Lp(a) OR = 1.001, 95% CI 1.000–1.001, p = 0.008; LVEF OR = 0.959, 95% CI 0.943–0.975, p<0.001 and HbA1C OR = 1.216, 95% CI 1.114–1.327, p<0.001). Furthermore, as shown in Table 4, our data demonstrated that the tertiles of plasma fibrinogen level was positively associated with an increased prevalence and severity of coronary atherosclerosis (assessed by the extent of stenosis or high GS) in a dose-dependent manner.

**Table 3:** Regression analyses to determine the independent predictor of high Gensini Score.

Variables	Univariate			Multivariate		
	OR	95%CI	p-value	OR	95%CI	p-value
Fibrinogen	1.294	1.144–1.463	**<0.001**	1.275	1.082–1.502	**0.004**
ESR	1.017	1.008–1.025	**<0.001**	1.023	1.012–1.035	**<0.001**
WBCC	1.068	1.010–1.130	**0.021**	1.038	0.975–1.106	0.241
Lp(a)	1.001	1.000–1.001	**0.005**	1.001	1.000–1.001	**0.008**
LVEF	0.961	0.946–0.977	**<0.001**	0.959	0.943–0.975	**<0.001**
HbA1C	1.219	1.125–1.322	**<0.001**	1.216	1.114–1.327	**<0.001**

Logistic regression analyses are performed. Bold values indicate statistical significance. Co-variates are included age, gender, BMI, current smoking, hypertension, family history of CAD, fasting blood glucose, TG, LDL-C, HDL-C and hs-CRP. ESR =  Erythrocyte sedimentation rate; WBCC =  White blood cell count; LP(a) =  Lipoprotein(a); LVEF  =  Left ventricular ejection fraction; HbA1c =  Glycosylated hemoglobinA1C; BMI =  Body mass index; CAD =  Coronary artery disease; TG =  Triglyceride; LDL-C =  Low-density lipoprotein-cholesterol; HDL-C =  High-density lipoprotein-cholesterol; hs-CRP =  High sensitivity C-reactive protein.

**Table 4:** Regression analyses to assess the severity of coronary atherosclerosis according to fibrinogen tertiles.

	Univariate			Multivariate		
	OR	95%CI	p-value	OR	95%CI	p-value
**The prevalence of coronary atherosclerosis**						
Fibrinogen	1.581	1.331–1.879	**<0.001**	1.476	1.178–1.851	**0.001**
Fibrinogen tertiles			**<0.001**			**0.001**
Tertile 1	1			1		
Tertile 2	1.217	0.935–1.584	0.144	1.192	0.889–1.598	0.241
Tertile 3	2.254	1.669–3.043	**<0.001**	2.003	1.383–2.903	**<0.001**
**The severity of coronary atherosclerosis**						
**Stenosis ≥50%**						
Fibrinogen	1.525	1.319–1.763	**<0.001**	1.674	1.384–2.024	**<0.001**
Fibrinogen tertiles			**<0.001**			**<0.001**
Tertile 1	1			1		
Tertile 2	1.262	1.002–1.590	**0.048**	1.304	1.011–1.683	**0.041**
Tertile 3	2.003	1.563–2.568	**<0.001**	2.182	1.599–2.978	**<0.001**
**Stenosis ≥ 75%**						
Fibrinogen	1.538	1.356–1.746	**<0.001**	1.638	1.388–1.933	**<0.001**
Fibrinogen tertiles			**<0.001**			**<0.001**
Tertile 1	1			1		
Tertile 2	1.128	0.917–1.388	0.254	1.112	0.887–1.395	0.365
Tertile 3	1.896	1.526–2.355	**<0.001**	1.939	1.484–2.533	**<0.001**
**High GS**						
Fibrinogen	1.294	1.144–1.463	**<0.001**	1.275	1.082–1.502	**0.004**
Fibrinogen tertiles			**<0.001**			**0.006**
Tertile 1	1			1		
Tertile 2	1.156	0.905–1.476	0.245	1.079	0.833–1.397	0.565
Tertile 3	1.595	1.262–2.016	**<0.001**	1.524	1.155–2.011	**0.003**

Logistic regression analyses are used. Bold values indicate statistical significance. The adjusting known confounders include age, gender, BMI, current smoking, hypertension, family history of CAD, fasting blood glucose, TG, LDL-C, HDL-C and hs-CRP. BMI =  Body mass index; CAD =  Coronary artery disease; TG =  Triglyceride; LDL-C =  Low-density lipoprotein-cholesterol; HDL-C =  High-density lipoprotein-cholesterol; hs-CRP =  High sensitivity C-reactive protein; GS =  Gensini score.

### Utility of fibrinogen in predicting the severity of coronary atherosclerosis

**Figure 3:**
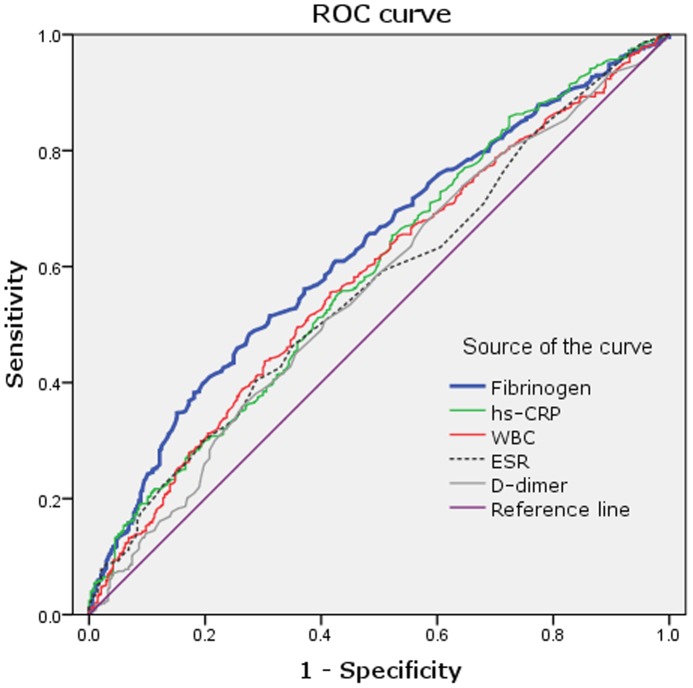
The cut-off value of fibrinogen and other non-specific inflammatory markers (hs-CRP, WBCC, ESR and D-dimer) in predicting the severity of coronary atherosclerosis. hs-CRP =  High sensitivity C-reactive protein; WBCC =  White blood cell count; ESR =  Erythrocyte sedimentation rate.

**Figure 4:**
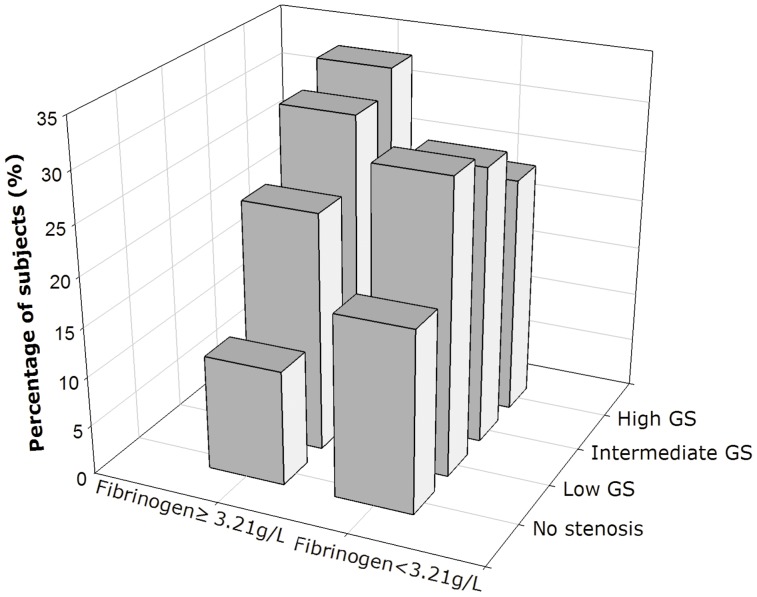
The distribution of subjects in high (≥3.21 g/L) or low (<3.21 g/L) fibrinogen groups according to the coronary stenosis and Gensini score.

Area under the ROC curves (AUC) indicated a well discriminatory power of plasma fibrinogen (AUC = 0.63, 95% CI 0.59–0.67, p<0.001) in predicting the severity of coronary atherosclerosis (Figure 3). The optimal cut-off value was 3.21 g/L (sensitivity of 52% and specificity of 69%). The predictive values of other non-specific inflammatory markers (hs-CRP, WBCC, ESR, or D-dimer) were also indicated in Figure 3. The value of fibrinogen in predicting the severity of coronary atherosclerosis was even superior to that of hs-CRP and WBCC. Using this cut-off value, we additionally divided the subjects into the two groups (high fibrinogen group ≥3.21 g/L and low fibrinogen group <3.21 g/L). As shown in Figure 4, the high fibrinogen group tended to have more severe coronary atherosclerosis and low percentage of no stenosis compared to the low fibrinogen group.

## Discussion

To the best of our knowledge, this is the first study evaluated the relationship of plasma fibrinogen level with the presence and severity of new-onset coronary atherosclerosis assessed by GS system in a large cohort of Han Chinese population. We found that the plasma fibrinogen level was positively associated with the presence and severity of coronary atherosclerosis in a dose-dependent manner after adjusting for a variety of conventional cardiovascular risk factors. Moreover, the ROC curve inferred a fibrinogen cut-off value of 3.21 g/L in predicting the severity of coronary stenosis. Therefore, the present study suggested a predictive value of baseline plasma fibrinogen level in Han Chinese patients with new-onset coronary atherosclerosis.

Fibrinogen is a soluble glycoprotein with a molecular weight of 340 kDa, which consists of three polypeptide chains. It is the precursor of fibrin and acts as a pivotal determinant of blood viscosity, platelet aggregation, fibrin formation, subsequent coagulation activation and fibrinolysis [20]. More recent work has shown that as fibrinogen level increased above physiologic levels, the balance between clotting and fibrinolysis is shifted toward the former [21]. Besides, fibrinogen has also been identified as an important inflammatory marker in the low grade inflammatory status of atherosclerosis [22]. Several lines of evidence implied that fibrinogen participated directly in atherogenesis [23]. During the last decades, numerous epidemiological investigations as well as meta-analysis have studied the relationship between plasma fibrinogen level and coronary atherosclerosis, while controversial results were generated.

The Strong Heart Study enrolled 2671 American Indians (with an average follow-up of 50 months) without clinical evidence of CAD at baseline suggested that fibrinogen could predict cardiovascular events independent of traditional risk factors. More importantly, this association was proved to be additional to established preclinical cardiovascular diseases [24]. However, the Fibrinogen Studies Collaboration analyzed 31 prospective studies in 154,211 adults without known CAD and reported that the association of fibrinogen level with CAD was reduced substantially after adjustment for several established cardiovascular risk factors [10]. Similarly, the Atherosclerosis Risk in Communities study suggested that in adults without history of CAD, the relationship of fibrinogen with the risk of recurrent CAD events was not statistically significant after adjusting for the potential risk factors [25]. In a recent study, fibrinogen was revealed to be an independent correlate of cardiovascular mortality among 13,195 patients with CAD, but could not provide additional prognostic information on top of that provided by traditional cardiovascular risk factors [11].

In the present study, we consecutively enrolled 2288 new-onset subjects referred to elective coronary angiography with angina-like chest pain and found that higher fibrinogen level was positively associated with the presence and severity of new-onset coronary atherosclerosis. Additionally, our data confirmed that plasma fibrinogen level was positively associated with other traditional cardiovascular risk factors, such as hs-CRP, TC, and LDL-C, which were in accordance with previous studies [26]. More interestingly, after adjusting for the known confounding factors, the association between fibrinogen and coronary atherosclerosis remained significant in the present study. Furthermore, our data suggested that a cut-off value of 3.21 g/L may be useful in predicting the severity of coronary stenosis. The findings were in consistent with previous studies among Western population [27] and our recent study in Chinese patients with CAD and diabetes mellitus [28]. However, those previous studies were limited by the lack of strict assessment system of coronary atherosclerosis or the relatively small sample size. In the present study, we enrolled a large cohort of subjects with new-onset coronary atherosclerosis instead of patients with chronic CAD or acute coronary syndrome and assessed coronary stenosis quantitatively by GS system. Finally, to the best of our knowledge, it is the first large sample study representative of Han Chinese population. Thus, our data confirmed and extended previous studies.

Our study has several limitations. First, our data cannot identify the causal relationship between plasma fibrinogen level and coronary atherosclerosis due to the cross-sectional design. Second, whether such association is present in other population should be studied in the future. Finally, we did not track the major adverse cardiovascular events of this population in the present study.

## Conclusions

In summary, the current data has indicated that higher fibrinogen level is positively and independently associated with the presence and severity of new-onset coronary atherosclerosis and the value of plasma fibrinogen >3.21 g/L suggests a more severe coronary stenosis.
